# Trained Immunity Attenuates Bleomycin-Induced Pulmonary Fibrosis by Promoting AMPK-Mediated Autophagy in Alveolar Macrophages

**DOI:** 10.3390/biology15161366

**Published:** 2026-08-11

**Authors:** Xinru Wang, Xinya Guo, Huiwen Meng, Zhiheng Sun

**Affiliations:** 1 Institute of Biomedical Science, College of Life Science, Henan Normal University, Xinxiang 453007, China; wangxinru20228@stu.htu.edu.cn (X.W.); 2404283130@stu.htu.edu.cn (X.G.); menghuiwen@stu.htu.edu.cn (H.M.); 2 State Key Laboratory of Cell Differentiation and Regulation, Henan Normal University, Xinxiang 453007, China

**Keywords:** trained immunity, alveolar macrophages, pulmonary fibrosis, autophagy, AMPK, mitochondria

## Abstract

Trained immunity (TI) represents a form of immune memory in innate immune cells, driven by sustained epigenetic and metabolic reprogramming that potentiates innate immune responses. Idiopathic pulmonary fibrosis (IPF) is a fatal interstitial lung disease characterized by persistent alveolar injury and pathological tissue remodeling. Given the central role of macrophages in IPF pathogenesis, we hypothesized that inducing TI could functionally reprogram these cells and attenuate fibrosis. In a murine model of pulmonary fibrosis induced by bleomycin, prior induction of TI via β-glucan enhanced autophagic activity in macrophages and reduced pathological collagen deposition. This trained response restricted bleomycin-triggered mitochondrial DNA release and suppressed the mitochondrial apoptosis pathway, thereby promoting macrophage survival. The protective effects were diminished by administration of the AMPK inhibitor Compound **C**. Our findings indicate that TI promotes mitophagy correlating with the AMPK-ULK1 signaling axis, thereby reducing alveolar macrophage apoptosis and uncovering a potential therapeutic strategy for pulmonary fibrosis.

## Simple Summary

This study aims to investigate whether TI alleviates IPF by regulating autophagy in alveolar macrophages. β-glucan was applied to induce TI, combined with a bleomycin-induced mouse model and a TGF-β-stimulated macrophage model. The results reveal that TI activates the AMPK-mTOR-ULK1 signaling axis, enhances mitophagy, reduces mitochondrial DNA release and macrophage apoptosis, and markedly decreases collagen deposition and the severity of fibrosis. Administration of the AMPK inhibitor Compound **C** reverses all the above protective effects. In conclusion, TI exerts anti-fibrotic effects by promoting AMPK-mediated autophagy to optimize mitochondrial quality control. This study provides a novel therapeutic strategy based on metabolic immune regulation for IPF, with potential value for clinical translation.

## 1. Introduction

Immunological memory, traditionally associated with the adaptive immune system due to repeated exposure to pathogens or their components, forms the basis of vaccine efficacy. Innate immune cells were long considered incapable of retaining a memory-like phenotype. However, emerging evidence indicates that invertebrates, lower vertebrates, and plants can mount adaptive responses to recurrent infections despite lacking the memory features of the adaptive immune system. These observations have led to the proposal of a new concept termed “trained immunity (TI)” [1]. TI is mediated through transcriptional, metabolic, and epigenetic reprogramming of monocytes or tissue macrophages following initial pathogen exposure, leading to enhanced pathogen clearance upon secondary challenge [2]. A variety of biological agents, including the fungal polysaccharide β-glucan, are capable of inducing TI. This phenomenon has been explored for potential applications in health and disease, particularly in inflammatory disorders and cancer [3]. To explore the critical downstream mediators through which TI exerts regulatory effects on pulmonary fibrosis, we analyzed the public dataset with the GEO accession number GSE57106 and identified ULK1 as one of the significantly upregulated genes. Nevertheless, the regulatory mechanism linking ULK1 to TI in pulmonary fibrosis remains elusive to date, and this mechanism may offer potential strategies for improving the treatment of pulmonary fibrosis in future research.

IPF is a progressive and fatal interstitial lung disease of unknown etiology, characterized by irreversible scarring of lung tissue that ultimately leads to respiratory failure [4]. Defective phagocytic function in macrophages is increasingly recognized as a critical link between epithelial injury and repair [5]. Alveolar macrophages from IPF patients exhibit a profound impairment in their ability to clear apoptotic cells, leading to the accumulation of cellular debris at sites of injury. This, in turn, promotes the release of pro-fibrotic mediators and damage-associated molecular patterns (DAMPs), fostering a pro-fibrotic microenvironment [6].

Autophagy serves as a crucial cytoprotective mechanism [7]. It is a dynamic process involving multiple molecular pathways that collectively maintain intracellular homeostasis. Selective autophagy serves as a core quality-control mechanism, operating through a “tag-receptor-LC3” molecular bridge—where receptor proteins such as p62 and OPTN recognize ubiquitinated substrates and tether them to the autophagosomal membrane—to precisely eliminate damaged organelles or aberrant protein aggregates. Beyond preserving organellar function, selective autophagy also directly modulates cellular signaling by degrading key signaling components, as exemplified by the endoplasmic reticulum autophagy receptor FAM134C associated with degradation of BMPRIA, thereby playing critical roles in diverse pathophysiological contexts including Parkinson’s disease and infection immunity [8,9]. Mitophagy, the selective clearance of damaged or superfluous mitochondria, represents another vital quality-control mechanism essential for maintaining alveolar macrophage homeostasis [10]. Mitophagy receptor pathways—associated with mitochondrial membrane proteins such as BNIP3, NIX, and FUNDC1—directly interact with LC3 to initiate autophagy. This process is tightly regulated by the AMPK-ULK1 signaling axis: AMPK not only activates general autophagy by inhibiting mTOR but also directly phosphorylates mitophagy-related proteins to enhance the specificity and efficiency of mitochondrial clearance. Emerging evidence indicates that mitophagy in alveolar macrophages suppresses NLRP3 inflammasome activation by removing damaged mitochondria and modulates the apoptotic threshold [11]. Moderate mitophagy helps sustain cellular homeostasis by eliminating dysfunctional mitochondria [12]. TI involves metabolic and epigenetic reprogramming that depends on energy and biomolecular precursors supplied by a maintained healthy mitochondrial network, changing concurrently with mitophagy. Nevertheless, few studies have reported the effects of TI on mitophagy.

AMPK (AMP-activated protein kinase), a central regulator of cellular energy metabolism, plays a critical role in the pathogenesis of pulmonary fibrosis [13]. Once activated, AMPK directly suppresses key pro-fibrotic signaling pathways. For instance, allicin—a compound derived from garlic—has been shown to activate AMPK, leading to inhibition of the TGF-β/Smad3 axis and resulting in a reduction in the expression of fibroblast activation markers such as α-SMA and fibronectin [14]. In another mechanism, CAMKK2 activates the AMPK/PGC-1α pathway, restoring mitochondrial dynamics by promoting fusion proteins MFN1/MFN2 and suppressing the fission protein DRP1, thereby improving mitochondrial function and suppressing both fibroblast activation and extracellular matrix production [15].

BLM-induced pulmonary fibrosis serves as a classic experimental model for investigating idiopathic pulmonary fibrosis. At present, the regulatory effect of β-glucan-triggered TI on pulmonary fibrosis remains unclear. Moreover, systematic verification is still lacking regarding whether TI intervention ameliorates pulmonary fibrosis by modulating mitophagy in alveolar macrophages, as well as its precise molecular regulatory mechanisms.

Given the above background, this study first established a BLM-induced pulmonary fibrosis model in C57BL/6 mice to systematically evaluate the regulatory effects of distinct interventions on BLM-triggered autophagy in alveolar macrophages. We focused on characterizing the altered profiles of autophagy and apoptosis in alveolar macrophages and further compared the pathological morphological differences in fibrotic lung tissues with or without TI treatment. Meanwhile, molecular biological techniques including ELISA and Western blot were utilized to detect inflammatory levels and the protein expression of autophagy-related signaling pathways in lung tissues from each group. Ultimately, this study aims to elucidate that TI regulates autophagy in alveolar macrophages via the AMPK-mTOR-ULK1 signaling axis and confirm its protective effect against BLM-induced pulmonary fibrosis in mice, thereby providing novel research perspectives and potential therapeutic targets for mechanistic studies of pulmonary fibrosis.

## 2. Materials and Methods

### 2.1. Reagents and Antibodies

β-Glucan was purchased from Invivogen Biotechnology (Hong Kong, China). TGF-β was obtained from R&D Systems (Minnesota, MN, USA). BLM was purchased from Sigma-Aldrich Company (Missouri, MO, USA). Antibodies against p-AMPKα (#AF3423), p-mTOR (#AF3308), and p-ULK1 (#AF4387) were sourced from Affinity (Changzhou, China). Antibodies targeting AMPKα (10929-2-AP), mTOR (66888-1-Ig), ULK1 (20986-1-AP), COL1A1 (67288-1-Ig), α-SMA (14395-1-AP), Beclin1 (66665-1-Ig), ATG5 (10181-2-AP) and β-actin (81115-1-RR) were procured from Proteintech (Wuhan, China). Compound **C** was purchased from MedChemExpress (Monmouth Junction, NJ, USA). The LC3B antibody (83506) was obtained from Cell Signaling Technology (Boston, MA, USA). The secondary antibodies Goat Anti-Rat (115-035-003) and Goat Anti-Rabbit (111-035-003) were both obtained from Jackson ImmunoResearch (Philadelphia, PA, USA).

### 2.2. In Vivo

#### 2.2.1. Mice

All experiments used 8-to-10-week-old C57BL/6 mice housed under specific pathogen-free (SPF) conditions at the Henan Normal University. Mice were maintained under a 12 h light/dark cycle with stable temperature and humidity and provided with sterile rodent chow and water ad libitum. All animal procedures were conducted in accordance with the National Institutes of Health Guide for the Care and Use of Laboratory Animals and approved by the Institutional Animal Care and Use Committee of the Henan Normal University (Proposal Number: HNSD-2025-04-29, Proposal Date: 2 March 2025).

#### 2.2.2. Animal Model

In vivo, 1 mg β-glucan was applied to establish the TI model [16]; bleomycin (BLM) at a dose of 2 U/kg (Missouri, MO, USA, B5507) was used to construct the pulmonary fibrosis model [17]; and Compound **C** was intraperitoneally injected at 25 mg/kg [18]. Mice were divided into two batches of experimental groups: the first batch contained the CON group, BLM group, and β-glucan + BLM group; the second batch contained the CON group, β-glucan + BLM group, and β-glucan + BLM + Compound **C** group. Each group included *N* = 3 mice, and all animal models were established in separate batches, resulting in six experimental groups with a total of 18 mice.

Treatment regimens for each group were standardized with vehicle controls: when mice in the TI group received an intraperitoneal injection of 1 mg β-glucan, mice in all other groups were administered an equal volume of 1 × PBS (Servicebio, Wuhan, China) intraperitoneally. Similarly, when 2 U/kg BLM was injected into mice of the BLM and TI combined groups, all other groups received an equivalent volume of 1 × PBS via intraperitoneal injection. The identical vehicle control strategy was adopted for Compound **C** administration: groups not receiving the inhibitor were injected with an equal volume of 1 × PBS intraperitoneally when the 25 mg/kg Compound **C** treatment was performed.

To induce trained immunity, mice were intraperitoneally injected with 1 mg β-glucan particles on Day-7. On Day 0, all relevant mice received intraperitoneal administration of 2 U/kg BLM. The body weight of each mouse was observed and recorded thereafter. On Day 21, all mice were anesthetized with isoflurane (Humanwell Pharmaceutical Co., Ltd., Yichang, China) and sacrificed by euthanasia. Bronchoalveolar lavage fluid (BALF) was collected first, followed by lung tissue harvesting. The left lung was immediately immersed in 4% paraformaldehyde (Servicebio, Wuhan, China) for subsequent embedding, while the right lung was transferred into centrifuge tubes and preserved in liquid nitrogen for subsequent extraction of tissue protein and RNA.

#### 2.2.3. H&E Staining

After sampling, fresh lung tissue was fixed with 4% paraformaldehyde (PFA) and then embedded with paraffin (Servicebio, Wuhan, China). The paraffin blocks on the tissue embedding box were cut into thin slices of 5 μm. After baking at 60 °C, the slices are gradually dewaxed and rehydrated and then soaked in distilled water to prevent cracking. First, the slices were stained with hematoxylin and then rinsed under running water. The sections were differentiated in HCl ethanol solution for 2 s, then flushed blue with tap water and stained with eosin (Servicebio, Wuhan, China). Subsequently, xylene (Servicebio, Wuhan, China) and ethanol (Servicebio, Wuhan, China) are dehydrated again. Finally, the stained sections are sealed with neutral resin (Servicebio, Wuhan, China).

#### 2.2.4. Masson’s Trichrome Staining

After deparaffinization and rehydration, the tissue sections were stained with iron hematoxylin staining solution (Servicebio, Wuhan, China) for 8 min. Following water washing, differentiation was performed with acidic ethanol differentiation solution for 15 s, followed by 1 min of water rinsing. The sections were then blued in Masson’s bluing solution (Servicebio, Wuhan, China) for 5 min and rinsed with water for 1 min. After another round of washing, sections were stained with Ponceau-fuchsin solution (Servicebio, Wuhan, China) for 5 min and washed with weak acid working solution (Servicebio, Wuhan, China) for 1 min. Subsequently, the sections were rinsed with phosphomolybdic acid working solution (Servicebio, Wuhan, China) for 1 min, followed by another 1 min wash with weak acid working solution. Finally, the sections were stained with aniline blue staining solution (Servicebio, Wuhan, China) for 2 min and rinsed with weak acid working solution for 1 min. The stained sections underwent ethanol dehydration and three cycles of xylene clearing before being mounted with neutral balsam (Servicebio, Wuhan, China).

#### 2.2.5. Bronchoalveolar Lavage (BAL) Fluid Collection

After tracheal exposure, an indwelling needle was inserted into the trachea, and the lungs were lavaged with 0.8 mL PBS. The recovered fluid was centrifuged at 500× *g* for 10 min, and the supernatant was stored for further analysis.

#### 2.2.6. Hydroxyproline Assay

Hydroxyproline content in the right lung was quantified using a hydroxyproline detection kit (MAK008, Sigma, Missouri, MO, USA) according to the manufacturer’s instructions.

#### 2.2.7. Immunohistochemistry

Paraffin-embedded lung tissues were first baked in an oven at 37 °C for 30 min, then cooled to room temperature. Deparaffinization was conducted by sequential immersion in xylene, absolute ethanol, 95% ethanol, 90% ethanol, 80% ethanol and 70% ethanol. Antigen retrieval was performed using citrate buffer for 10 min, followed by cooling to room temperature. Sections were rinsed three times with double-distilled water, then blocked with peroxidase-blocking solution for 10 min and washed three times again. Non-specific binding was blocked with immunohistochemical blocking buffer at 37 °C for 15 min, after which the corresponding primary antibody was applied and incubated at 4 °C overnight. On the next day, sections were washed three times and incubated with the matching secondary antibody at room temperature for 2 h, followed by three washes. Tertiary antibody incubation was carried out for 40 min, then the slides were washed three times again. DAB staining was performed for 5 min under light protection, and staining intensity was checked under a light microscope. Counterstaining with hematoxylin lasted for 2 s, followed by bluing treatment with bluing solution, and another microscopic observation. Serial dehydration was completed via 70% ethanol, 80% ethanol, 90% ethanol, 95% ethanol, absolute ethanol and xylene in sequence. Finally, the sections were mounted with neutral resin and air-dried. All reagents used for immunohistochemistry were purchased from Beyotime Biotechnology (Shanghai, China).

#### 2.2.8. Body Weight Monitoring of Animals

Starting from Day 0 of the experiment (the day of intratracheal instillation of bleomycin), each mouse was weighed using a calibrated electronic balance every two days (on Day 0, 2, 4, 6, 8, 10, 12, 14, 16, 18 and 21), and body weight data were recorded in detail. All weighing operations were performed by the same experimenter to guarantee consistent manipulation. All body weight values were recorded in grams (g). Data of each group were presented as mean ± standard deviation (mean ± SD). One-way analysis of variance (one-way ANOVA) was adopted for intergroup comparisons to evaluate the effect of TI treatment on the body weight changes in mice.

### 2.3. In Vitro

#### 2.3.1. Cell Culture and Models

The RAW264.7 macrophage cell line was obtained from the Type Culture Collection of the Chinese Academy of Sciences (Shanghai, China). Cells were cultured in phenol DMEM supplemented with 10% fetal bovine serum (Tianhang Biotechnology, Deyang, China) and 1% penicillin–streptomycin (Biosharp, Hefei, China) at 37 °C in a 5% CO_2_ atmosphere. Cells were used within five passages and seeded at ~85% confluency for experiments.

For vitro TI, RAW264.7 cells were treated with 5 μg/mL β-glucan for 24 h, washed with PBS, and rested in fresh medium for 5 days. A fibrotic model was established using 10 ng/mL TGF-β (R&D Systems, Minneapolis, MN USA). Where indicated, 10 μM Compound **C** (MCE, Monmouth Junction, NJ, USA) was applied.

#### 2.3.2. Quantitative PCR

Total RNA was extracted from tissues and cells using Trizol reagent (Servicebio, Wuhan, China). Subsequently, 1 μg of RNA was reverse-transcribed using HiScript II Reverse Transcriptase (Vazyme, Nanjing, China). The resulting complementary DNA was utilized for real-time PCR amplification on a Fast RealTime PCR System (Roche Diagnostics, Rotkreuz, Switzerland) using Hieff qPCR SYBR Green Master Mix (Yeasen Biotechnology, Shanghai, China). The oligonucleotide primers used for qPCR amplification are listed in Table 1. The relative expression levels of the target genes were determined using the 2^−ΔΔCt^ method. All experiments were performed with three replicates. β-actin (Actb) was used as the reference gene for quantitative PCR assays in this study. All primers targeting the target genes and the reference gene applied in the present work were independently designed and synthesized by a biotechnology company (Sangon Biotech, Shanghai, China) based on the published mouse reference sequences from the NCBI GenBank database. The specificity of each primer pair was strictly verified via melting curve analysis. Primer sequences are provided in Table 1.

#### 2.3.3. Western Blotting

Proteins were obtained from tissues and cells using RIPA buffer (Beyotime Biotechnology, Shanghai, China). The protein lysates were separated on SDS-polyacrylamide gel (Beyotime Biotechnology, Shanghai, China) at a concentration of 8–15%. After electrophoresis, the proteins were transferred onto polyvinylidene fluoride polyethylene membranes (Millipore, Billerica, MA, USA) and then blocked with 5% BSA (Solarbio Science & Technology, Beijing, China) at room temperature for 1 h. The proteins were incubated overnight with the previously described specific primary antibody at 4 °C. β-actin or GAPDH were used as internal controls. Then, the membrane was rinsed with TBST buffer (Servicebio, Wuhan, China) and incubated at room temperature for 2 h with the enzyme-labeled secondary antibody. After three washes with TBST buffer, the protein bands were displayed in the Odyssey^®^XF imaging system (LI-COR Biosciences, Lincoln, NE, USA).

#### 2.3.4. Flow Cytometry

After the establishment of TI and pulmonary fibrosis models, cells cultured in six-well plates were harvested. The cell culture supernatant was first collected into centrifuge tubes, and adherent cells were gently detached with culture medium and transferred to the same tubes. Samples were centrifuged at 1000× *g* for 5 min, the supernatant was discarded, and the cell pellet was washed once with PBS followed by another round of centrifugation and supernatant removal. After all cell-washing steps, cells for single-stain compensation control tubes were resuspended in 1× binding buffer. Cells for the FITC single-stain tube were incubated at 56 °C for 10 min, and the reaction was terminated with 1× PBS. Cells for the PI single-stain tube were fixed with 4% paraformaldehyde on ice for 10 min, then centrifuged, and the supernatant was discarded, and cells were resuspended in 1× binding buffer. FITC reagent and PI reagent were separately added to the corresponding single-stain tubes under light protection. For the experimental group, the mixed solution of FITC and PI was added, followed by 5 min incubation away from light, and the staining reaction was terminated with PBS finally. All centrifuge tubes containing cell samples were placed on ice prior to detection via flow cytometry. All reagents used were purchased from Beyotime Biotechnology.

#### 2.3.5. Immunofluorescence

After alcohol cauterization, the slides were placed in 24-well plates. Then, the cultured cells were inoculated onto the slides and incubated in a CO_2_ incubator (Thermo Fisher Scientific, Waltham, MA, USA) for an appropriate period of time. After rinsing three times with PBS, the slides were fixed at 4% paraformaldehyde at room temperature for 15 min. After fixation, they were washed three times with PBS and incubate at room temperature in 0.3%TritonX-100 solution for 5 min to increase permeability. After penetration, the slides were washed three times with PBS. Subsequently, they were incubated at room temperature in 5% BSA for 1 h to reduce non-specific staining. The primary antibodies against F4/80, LC3B and AMPK were diluted in 5% BSA at a ratio of 1:100 and left overnight at 4 °C. Then, they were washed three times with PBS, the secondary antibody conjugated with Alexa Fluor 488 and Alexa Fluor647 at a dilution of 1:250 was bound, and the primary antibodies were incubated at room temperature for one and a half hours in the dark. After PBS washing, DAPI was used as the nuclear dye to stain the cell nuclei. Then, the PBS was washed three times, the antifluorescence quencher (Beyotime Biotechnology, Shanghai, China) was added and the plate was sealed. Finally, the slides were observed using a Nikon Eclipse Ti-U fluorescence microscope equipped with a DS-Ri1 digital camera (Nikon Corporation, Tokyo, Japan).

#### 2.3.6. Enzyme-Linked Immunosorbent Assay (ELISA)

Levels of IL-6 and TNF-α in cell supernatants or BAL fluid were measured using mouse ELISA kits (Solarbio, Beijing, China) following the manufacturer’s instructions.

#### 2.3.7. Alveolar Macrophage Isolation

Tissue-resident alveolar macrophages (AMs) were isolated from euthanized mice via bronchoalveolar lavage (BAL). The mouse trachea was surgically exposed, and an indwelling needle connected to a 1 mL prefilled syringe containing 1× PBS was inserted into the trachea. PBS buffer was gently instilled into the lung tissue; after 30 s of gentle lung massage, the syringe plunger was slowly withdrawn, and the recovered bronchoalveolar lavage fluid (BALF) was transferred to an ice-cold 15 mL conical centrifuge tube. This infusion–retrieval lavage cycle was repeated several times for each mouse. Collected BALF was centrifuged at 300× *g* for 10 min at 4 °C. The resulting cell pellet was resuspended in 5 mL Red Blood Cell Lysis Buffer (Beyotime Biotechnology, Shanghai, China) and incubated at room temperature for 5 min to lyse erythrocytes. The lysis reaction was terminated by adding 5 mL PBS, followed by another centrifugation at 300× *g* for 10 min at 4 °C. Harvested cells were resuspended in complete medium (DMEM supplemented with 10% fetal bovine serum (FBS) and 1% penicillin–streptomycin) and cultured at 37 °C under 5% CO_2_ constant temperature conditions.

#### 2.3.8. ECAR

Extracellular acidification rate (ECAR) was measured using a Seahorse XF96 Analyzer (Agilent Technologies, Santa Clara, CA, USA). Cells were seeded in XF96 plates (Agilent Technologies, Santa Clara, CA, USA), and assays were performed following injection of glucose (Agilent Technologies, Santa Clara, CA, USA), oligomycin (Agilent Technologies, Santa Clara, CA, USA), and 2-DG (Agilent Technologies, Santa Clara, CA, USA) according to the manufacturer’s protocol.

#### 2.3.9. Autophagic Flux

Alveolar macrophages and RAW264.7 cells were evenly seeded into 24-well plates (Biosharp, Beijing, China) to establish in vitro TI and pulmonary fibrosis models. The transfection mixture was prepared as follows: 1 μg of mRFP-GFP-LC3 plasmid (Genechem, Shanghai, China) was diluted in serum-free medium and mixed by gentle pipetting, followed by incubation at room temperature for 5 min. Separately, 1 μL Lipofectamine 3000 transfection reagent (Sigma, Missouri, MO, USA) was diluted in serum-free medium, gently mixed and incubated at room temperature for another 5 min. The two diluents were combined with soft pipetting during addition and incubated at room temperature for 20 min. The original culture medium in each well of the 24-well plate was aspirated, and cells were gently washed three times with serum-free medium. An appropriate volume of serum-free medium was supplemented to each well, followed by slow dropwise addition of the prepared transfection mixture. The culture plate was softly shaken to achieve uniform distribution of the mixture. After 4 h of incubation in a CO_2_ incubator, the transfection medium was discarded and replaced with complete medium containing 10% FBS for further 24 h culture. Subsequently, the culture medium was aspirated, and cells were gently rinsed three times with pre-cooled PBS buffer. Each well was filled with 4% paraformaldehyde fixative for 10 min of fixation at room temperature, then the residual fixative was removed. After three PBS washes, cell coverslips were mounted onto glass slides with anti-fade mounting medium (Beyotime Biotechnology, Shanghai, China), left at room temperature for 5 min for preliminary solidification of the mounting medium, stored at 4 °C away from light, and finally observed under a fluorescence microscope (Carl Zeiss AG, Oberkochen, Germany).

### 2.4. Statistical Analysis

The statistical analysis was performed using Prism (Prism 5 for Windows, GraphPad Software Inc., San Diego, CA, USA). A two-tailed Student’s *t*-test was conducted to analyze statistical significance between two groups. Data are shown as means ± SD. *p* < 0.05 was considered to be statistically significant.

## 3. Results

### 3.1. Autophagy Is Involved in BLM-Induced Pulmonary Fibrosis

**Figure 1:**
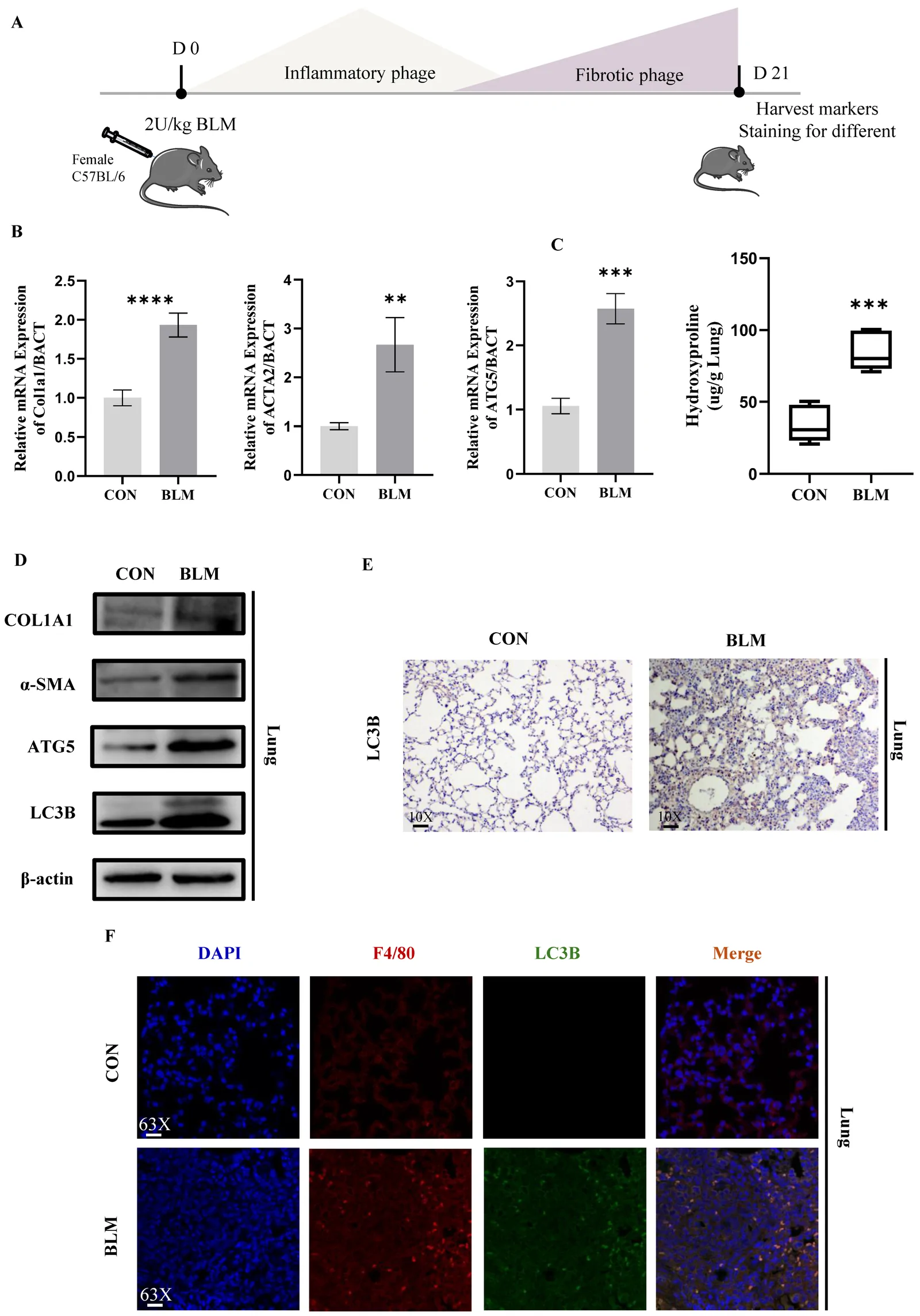
Autophagy involves BLM-induced pulmonary fibrosis. (**A**) In vivo pulmonary fibrosis models: A pulmonary fibrosis model was established using 2U/Kg bleomycin (*n* = 3/group). (**B**) The expression levels of Col1a1, ACTA2 and ATG5 mRNA in mouse lung tissues were detected by qPCR (*n* = 3) and compared with the normal saline group. (**C**) Hydroxyproline content in the lungs of mice in the normal saline group and the BLM group (*n* = 3) are shown. (**D**) Western blot was used to detect that the protein expressions of ATG5, LC3B, COL1A1 and α-SMA in the lung tissues of BLM-treated mice were increased (*n* = 3). (**E**) Immunohistochemical staining of LC3B in the lungs of mice in the normal saline group and the BLM group (*n* = 3) are shown. (**F**) The co-localization of alveolar macrophages F4/80 and autophagy LC3B in mouse lung tissue was shown by immunofluorescence (*n* = 3). Nucleus (blue), F4/80 (red), LC3B (green). Single dots correspond to individual mice. ** *p* < 0.01, *** *p* < 0.001, and **** *p* < 0.0001; paired Student’s *t*-test comparing BLM group and control group. The original Western blot images for (**D**) are provided in the File S1.

As a highly regulated cellular response to endogenous and exogenous stress, autophagy can be rapidly induced and is implicated in a wide range of physiological and pathological processes. To systematically investigate the dynamic role of autophagy in pulmonary fibrosis, we established a mouse model of IPF induced by bleomycin (BLM) (Figure 1A). Using qPCR analysis, we evaluated the expression of autophagy-related genes in mouse lung tissues. Notably, we observed a concurrent upregulation at the transcriptional level of the fibrosis markers Col1a1 and ACTA2, along with the autophagy-related gene ATG5, in the BLM-treated group compared with the control group (Figure 1B), suggesting a potential link between autophagy activation and fibrotic progression. Furthermore, hydroxyproline assay confirmed a significant increase in collagen deposition in BLM-exposed lungs (Figure 1C). To further examine changes in autophagy during fibrosis, we assessed a panel of autophagy-related proteins in lung tissues from the experimental model. Western blot analysis revealed increased protein expression of LC3-II and ATG5, consistent with the elevation of collagen and α-SMA in BLM-treated mice (Figure 1D). These protein-level changes aligned with the corresponding gene expression patterns. Immunohistochemical staining for LC3B further suggested its specific accumulation within fibrotic regions of BLM-treated lungs compared with controls (Figure 1E). Alveolar macrophages, the key resident immune cells in the alveolar space, contribute to pulmonary homeostasis changing concurrent with phagocytic clearance of harmful substances [7]. Immunofluorescence staining revealed co-localization of F4/80-positive alveolar macrophages with LC3B in BLM-treated lung tissues (Figure 1F), indicating that autophagy activation occurs predominantly in this cell type. Together, these results suggest that the autophagy pathway plays an important role in pulmonary fibrosis and exhibits cell type-specific activation.

### 3.2. TI Ameliorates BLM-Induced Pulmonary Fibrosis

TI enables cells to develop a memory-like phenotype, wherein their baseline functional state is durably altered. This reprogramming is likely extended to the basal activity of autophagy. Given the established role of autophagy in fibrosis, we further investigated the therapeutic potential of TI in regulating pulmonary fibrosis.

**Figure 2:**
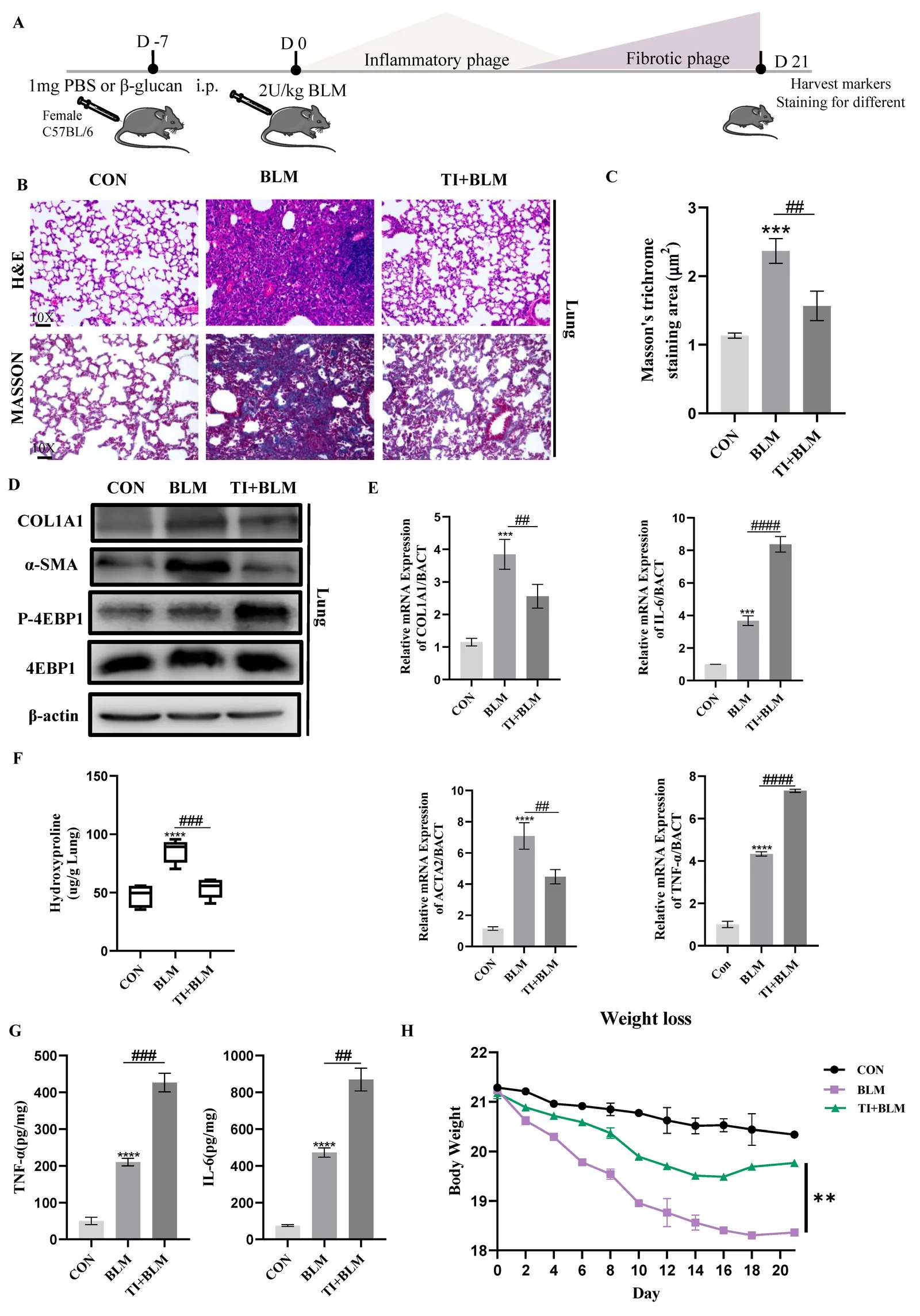
TI can alleviate pulmonary fibrosis induced by BLM. (**A**) In vivo TI and pulmonary fibrosis models: A pulmonary fibrosis model was established using 2U/Kg bleomycin, 1mg/Kg β-glucan or PBS (*n* = 3/group). (**B**,**C**) Masson tricolor staining and H&E staining of representative lung sections of mice in each group (*n* = 3) are shown. (**D**) Western blot detection showed that the protein expressions of COL1A1 and α-SMA in the lung tissues of mice treated with BLM + TI were both decreased (*n* = 3). (**E**) The mRNA expression levels of Col1a1, ACTA2, IL-6 and TNF-α in mouse lung tissues were detected by qPCR (*n* = 3) and compared with the BLM group. (**F**) Hydroxyproline content in the lungs of mice in the control group, BLM group and BLM + TI group (*n* = 3) are shown. (**G**) The contents of TNF-α and IL-6 in bronchoalveolar lavage fluid (BALF) of mice were determined by ELISA (*n* = 3). (**H**) Changes in mouse body weight are shown. Single dots correspond to individual mice. ** *p* < 0.01, *** *p* < 0.001, and **** *p* < 0.0001; paired Student’s *t*-test comparing BLM group and control group. ^##^ *p* < 0.01, ^###^ *p* < 0.001 and ^####^ *p* < 0.0001; paired Student’s *t*-test comparing between BLM group and TI + BLM group. The original Western blot images for (**D**) are provided in the File S1.

First, we established the mouse TI model by intraperitoneally injecting 1 mg β-glucan into mice on Day-7 and Day-4, respectively. On Day 0, mice received an intraperitoneal injection of bleomycin (BLM) at a dose of 2 U/kg to construct the idiopathic pulmonary fibrosis model [16,19]. Histopathological evaluation via H&E and MASSON staining revealed characteristic alveolar structural disruption and abnormal blue collagen deposition in the BLM-induced pulmonary fibrosis model. In contrast, β-glucan-induced TI significantly ameliorated both BLM-induced alveolar damage and collagen accumulation (Figure 2A). Quantitative analyses confirmed that TI reduced both the fibrotic area and alveolar destruction index (Figure 2B). At the molecular level, Western blot and qPCR analyses consistently indicated that TI effectively suppressed the expression of Col1a1 and ACTA2. Phosphorylated 4EBP1 (p-4EBP1 phosphorylated eukaryotic translation initiation factor 4E-binding protein 1) has been utilized as a functional marker for metabolic reprogramming in β-glucan-trained macrophages in previous studies [20]. Herein, we introduced p-4EBP1 as a molecular marker of TI to confirm the multiple typical characteristics of TI upon two rounds of β-glucan stimulation in our system. Western blot results revealed elevated expression of p-4EBP1 in the TI group. Concurrently, elevated expression of the pro-inflammatory cytokines TNF-α and IL-6 was observed (Figure 2C,D). Hydroxyproline content assay further validated that TI significantly alleviated excessive collagen deposition (Figure 2E).

TI is characterized by specific chemical modifications on histones—such as H3K4me3 and H3K27ac—that maintain chromatin in an “open” state at promoters of pro-inflammatory genes (e.g., those encoding IL-6 and TNF-α). Upon secondary stimulation, these genes undergo more rapid and robust transcription and translation, leading to enhanced cytokine release [21]. In this study, precise quantification of TNF-α in bronchoalveolar lavage fluid by ELISA provided key evidence for the mechanistic basis of TI. Results showed significantly increased secretion of TNF-α and IL-6 in the BLM + TI group (Figure 2F). This immunomodulatory pattern aligns with the distinct epigenetic reprogramming features of TI. Furthermore, TI effectively reversed the progressive weight loss induced by BLM (Figure 2G). In addition, we performed co-localization staining of F4/80 and LC3B on lung tissues from TI mice. The results indicated that TI markedly potentiated autophagy activity in alveolar macrophages compared with the BLM-only group (Figure S1). Collectively, these multi-level findings indicate that TI exerts multi-dimensional anti-fibrotic effects by coordinated modulation of immune responses and extracellular matrix remodeling.

### 3.3. TI Promotes Mitophagy and Attenuates Alveolar Macrophage Apoptosis In Vivo and In Vitro

**Figure 3:**
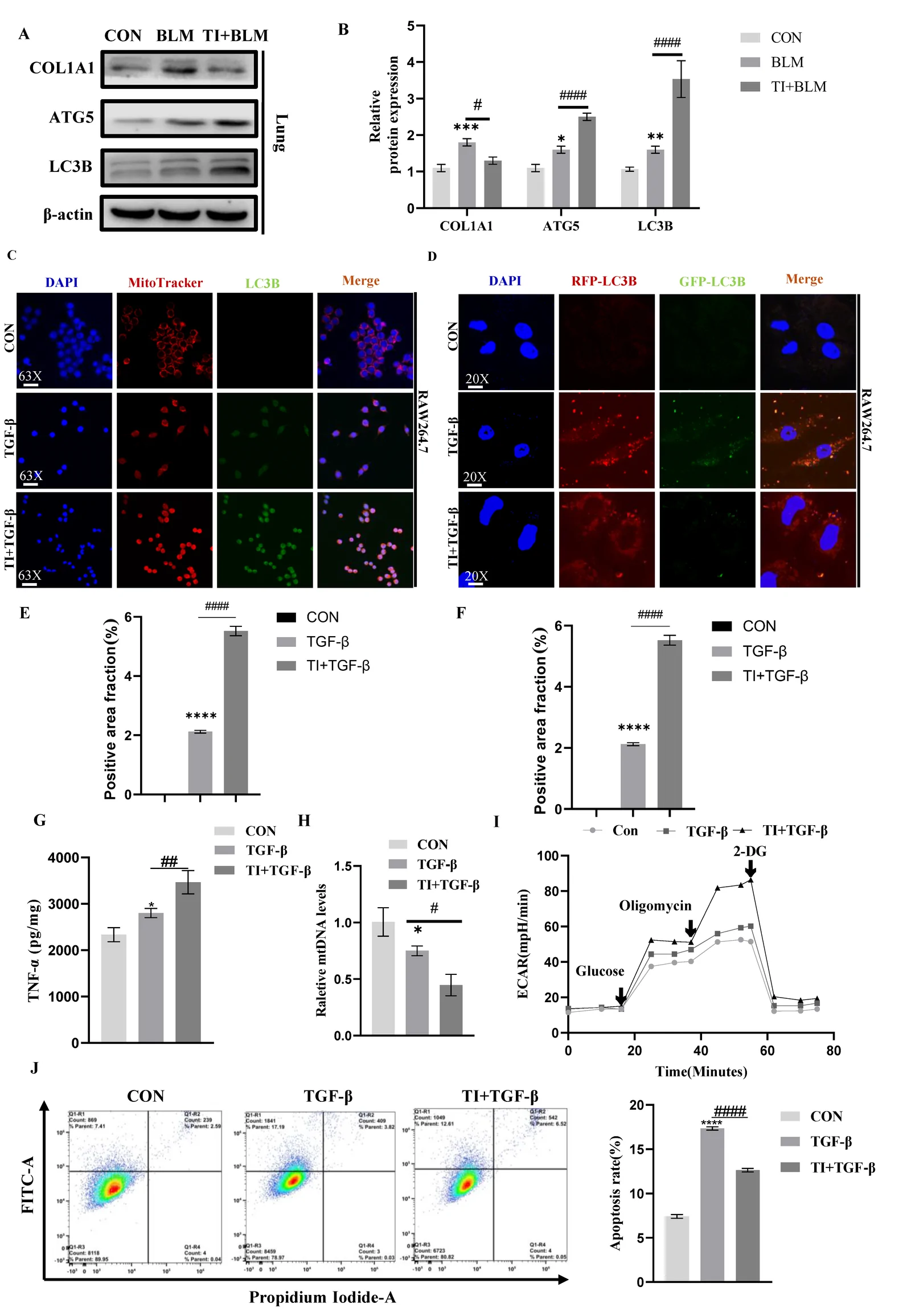
TI promotes mitochondrial autophagy both in vivo and in vitro and alleviates apoptosis of alveolar macrophages. (**A**,**B**) Western blot was used to detect the protein expressions of COL1A1, ATG5 and LC3B in RAW264.7 cells treated with TGF-β and TGF-β + TI. (**C**) Co-localization of mitochondrial and LC3B expression after trained immunization are shown; Nucleus (blue), MitoTracker (red), LC3B (green). (**D**) Detection of mitochondrial autophagy flux by immunofluorescence is shown; (**E**,**F**) quantification of the immunofluorescence-positive areas in (**D**,**E**) are shown. (**G**) The content of TNF-α in bronchoalveolar lavage fluid (BALF) of mice was determined by ELISA. (**H**) The expression level of mtDNA was detected by qPCR. (**I**) The ECAR of RAW264.7 cells under TI was detected by a hippocampal analyzer. Flow cytometry (**J**) showed that compared with the TGF-β alone treatment group, the apoptosis of RAW264.7 cells was reduced in the TI + TGF-β treatment group. Single dots correspond to individual mice. * *p* < 0.05, ** *p* < 0.01, *** *p* < 0.001 and **** *p* < 0.0001; paired Student’s *t*-test comparing TGF-β group and control group. ^#^ *p* < 0.05, ^##^ *p* < 0.01, and ^####^ *p* < 0.0001; paired Student’s *t*-test comparing between TGF-β group and TI + TGF-β group. The original Western blot images for (**A**) are provided in the File S1.

The core of TI lies in metabolic reprogramming, shifting from oxidative phosphorylation toward glycolysis. Mitochondria, serving as both energy hubs and signaling platforms, are critically involved in this process. Mitophagy, which selectively removes damaged or superfluous mitochondria, is essential for enabling metabolic adaptation. We therefore sought to determine the role of mitophagy in alveolar macrophage injury in vivo and in vitro. We first performed in vivo experiments to compare the effects of BLM alone versus BLM combined with TI on mouse mitochondria. Western blot analysis revealed that BLM + β-glucan treatment not only upregulated LC3B-II and ATG5 expression but also reduced collagen levels (Figure 3A,B), suggesting that TI enhances autophagy while attenuating collagen deposition and mitigating pulmonary fibrosis. In order to further confirm whether TI can enhance mitochondrial autophagy in vitro, we stimulated macrophages with the fibrotic factor TGFβ [22]. In vitro studies using dual fluorescence labeling with Mito Tracker and LC3B indicated that TI led to mitochondrial fragmentation and significant co-localization with LC3B-positive structures (Figure 3C), supporting enhanced mitophagy. Mitophagy is a complete dynamic process for selective mitochondrial clearance. We further detected mitophagy flux and confirmed that TI effectively augments mitophagy flux and maintains mitophagy in an activated state (Figure 3D). Consistently, elevated autophagic flux was also observed in alveolar macrophages (Figure S2). Quantitative analysis of immunofluorescence-positive regions yielded consistent results (Figure 3E,F). The coordinated changes in autophagic markers and mitochondrial dynamics suggest profound dysregulation of mitochondrial quality control during fibrotic progression. ELISA revealed that TI significantly elevated levels of relevant inflammatory cytokines (Figure 3G). Mitochondrial apoptosis is jointly regulated by mis-localized mtDNA and extracellular acidification rate (ECAR) dynamics, which integrate genetic stress and metabolic signals to determine cell fate. On one hand, qPCR-based quantification of cytosolic mtDNA showed increased levels in the TGF-β group compared with controls, which were reduced by TI (Figure 3H), providing direct evidence of mtDNA leakage due to mitochondrial stress. On the other hand, the accompanying metabolic shift-reflected by elevated ECAR and a glycolytic phenotype-like facilitates apoptosis execution by supplying energy substrates and lowering survival thresholds. Metabolic flux analysis further revealed that TI induced a unique reprogramming profile, characterized by enhanced glycolytic capacity and optimized oxidative phosphorylation (Figure 3I). Consistent with our previous in vitro findings, we confirmed that BLM increased macrophage apoptosis, whereas TI reduced the number of apoptotic cells. Quantitative analysis confirmed that β-glucan treatment significantly decreased the apoptosis rate (Figure 3J). Together, these results indicated that TI exerts a protective effect against BLM-induced alveolar macrophage injury and apoptosis, likely changing concurrently with the promotion of mitophagy.

### 3.4. TI Attenuates Pulmonary Fibrosis Primarily by Activating Autophagy via the AMPK-mTOR-ULK Signaling Axis

**Figure 4:**
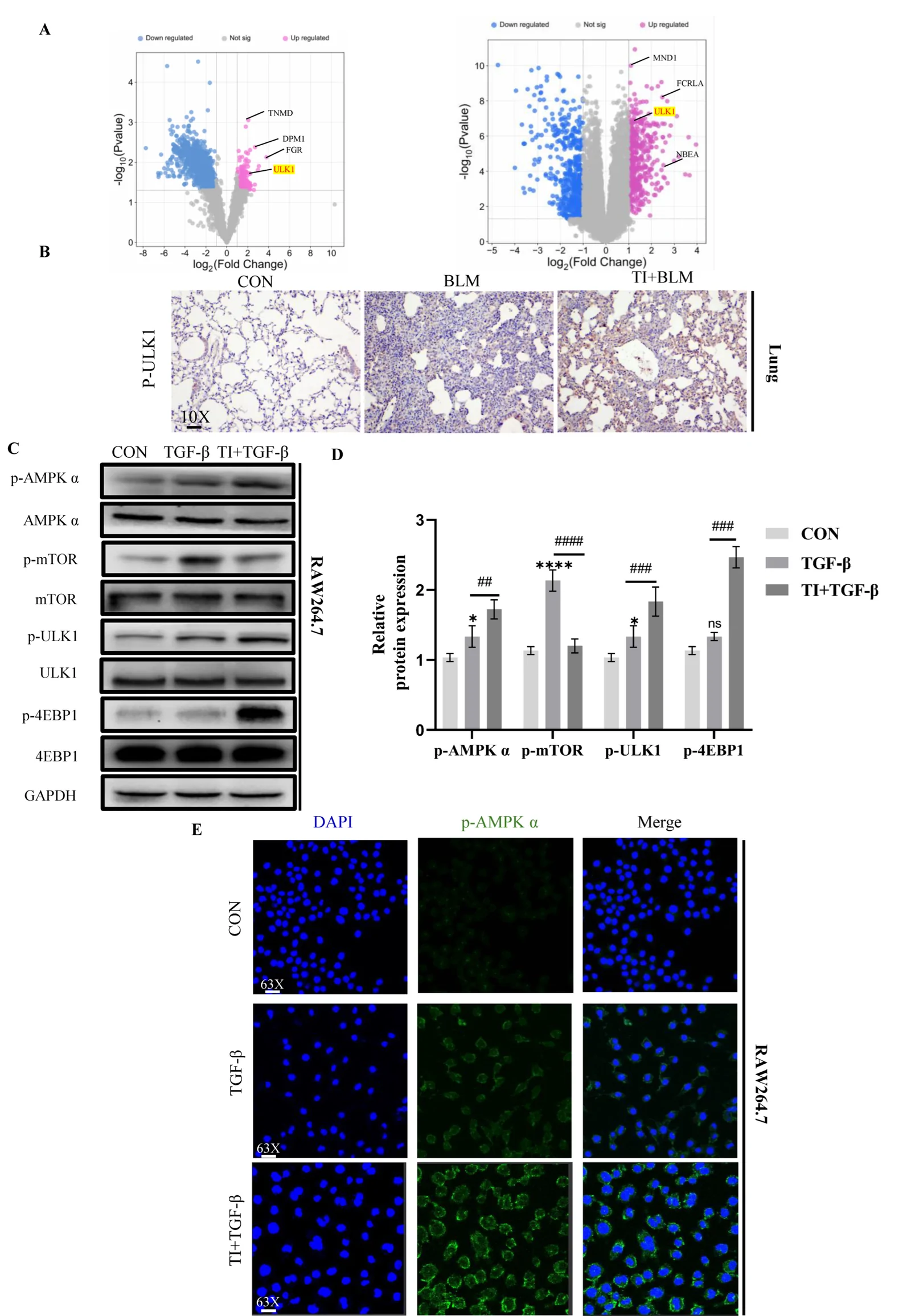
TI mainly activates autophagy through the AMPK-mTOR-ULK signaling pathway to inhibit pulmonary fibrosis. (**A**) Volcano map and heat map analysis revealed that the expression of ULK1 in TI and IPF human samples in GSE57206 and GSE47460 was significantly upregulated. (**B**) Immunohistochemical staining showed the expression of P-ULK in the lung tissue of mice. (**C**,**D**) Western blot was used to detect the protein expressions of P-AMPK, P-mTOR and P-ULK in RAW264.7 cells in the control group, TGF-β group and TI + TGF-β group. (**D**) The content of TNF-α in bronchoalveolar lavage fluid of mice was determined by ELISA. (**E**) Immunofluorescence showed that the expression of AMPK was significant after TI + TGF-β treatment, Nucleus (blue). ns (not significant), * *p* < 0.05, and **** *p* < 0.0001; paired Student’s *t*-test comparing TGF-β group and control group. ^##^ *p* < 0.01, ^###^ *p* < 0.001, and ^####^ *p* < 0.0001; paired Student’s *t*-test comparing between TGF-β group and TI + TGF-β group. The original Western blot images for (**C**) are provided in the File S1.

To further investigate the mechanisms by which TI regulate autophagy, we analyzed public datasets (GSE accession numbers GSE47460 and GSE57206). The results showed that compared to the control group, ULK1 was among the autophagy-related genes with the most pronounced upregulation in lung tissues stimulated by TI (Figure 4A). Subsequent immunohistochemical analysis revealed that fibrotic tissues from the BLM + β-glucan group not only exhibited reduced structural damage but also showed markedly elevated phosphorylation of ULK1, with specific accumulation in lesioned areas. This spatial heterogeneity in protein expression directly links bioinformatic predictions to actual pathological changes, confirming the pathway’s activation within the tissue microenvironment (Figure 4B). We then assessed the protein levels of p-AMPKα, AMPKα, p-mTOR, mTOR, p-ULK1, and ULK1 by Western blot. While no significant differences in p-AMPK or p-mTOR levels were observed between the control and TI groups, the TGF-β group showed lower p-AMPK and higher p-mTOR compared to both control and TI groups. In contrast, β-glucan treatment increased p-AMPKα expression. Grayscale quantification provided a quantitative profile of these protein levels. Furthermore, the expression of P-4EBP1 in the vitro TI group also increased (Figure 4C,D). This coordinated change across multiple protein nodes fully pointed towards the activation state of the AMPK/mTOR-autophagy and its synergy with TI. Furthermore, multicolor immunofluorescence imaging indicated that activated AMPKα (p-AMPKα) formed distinct punctate structures within the cytoplasm of F4/80^+^ macrophages in the TI group, forming characteristic signaling hubs in alveolar macrophages (Figure 4E). Furthermore, in mouse alveolar macrophages, these immunofluorescence signals were also enhanced in response to the stimulation of β-glucan (Figure S3). These results further corroborate the conclusion that TI activates autophagy in vivo. Collectively, our findings identify the AMPK-mTOR-ULK1 axis as a central signaling hub in TI, mediating its anti-fibrotic effects through multi-level regulation of autophagy activity.

### 3.5. Phosphorylation of AMPK-mTOR-ULK1 Pathway Proteins Regulates Macrophage Apoptosis in TI

**Figure 5:**
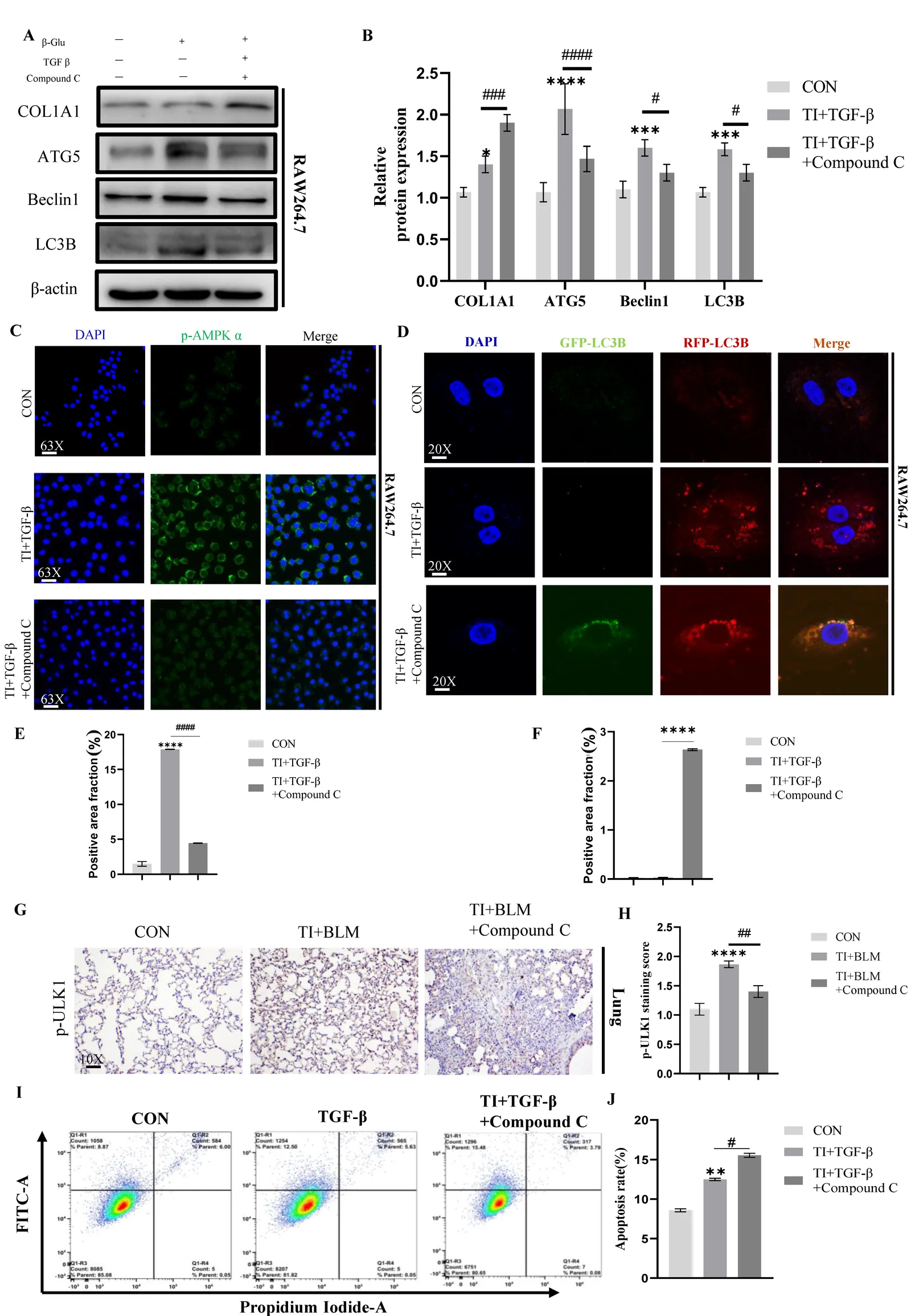
Phosphorylation of AMPK-mTOR-ULK1 pathway proteins affects macrophage apoptosis caused by TI. (**A**,**B**) The Western blot results showed that Compound **C** could increase the expression of COL1A1 and reduce the expression of autophagy-related markers. (**C**) Immunofluorescence showed that Compound **C** treatment could reduce the expression of AMPK. (**D**) Detection of mitochondrial autophagy flux by immunofluorescence is shown; (**E**,**F**) quantification of the immunofluorescence-positive areas in (**D**,**E**) is shown. (**G**,**H**) The immunohistochemical results showed that compared with the TI + BLM group, Compound **C** treatment reduced the expression of p-ULK1. (**I**,**J**) Compound **C** treatment can increase cell apoptosis. Single dots correspond to individual mice. * *p* < 0.05, ** *p* < 0.01, *** *p* < 0.001, and **** *p* < 0.0001; paired Student’s *t*-test comparing TI + TGF-β group and control group. ^#^ *p* < 0.05, ^##^ *p* < 0.01, ^###^ *p* < 0.001, and ^####^ *p* < 0.0001; paired Student’s *t*-test comparing between TI + TGF-β group and TI + TGF-β + Compound **C** group. The original Western blot images for (**A**) are provided in the File S1.

To determine whether TI modulates the TGF-β-induced fibrotic changing concurrent with AMPK-dependent autophagy activation, we employed the AMPK inhibitor Compound **C**. Western blot analysis revealed that, compared with the TI group without inhibitor, the Compound **C** treated group exhibited a marked increase in collagen levels, alongside reduced expression of the autophagy marker proteins ATG5, Beclin1, and LC3B. Densitometric quantification of Western blot bands confirmed significant differences between the two groups (Figure 5A,B). Multiplex immunofluorescence further showed a specific decrease in activated AMPK in inhibitor-treated cells (Figure 5C). Similarly, such elevated autophagic flux was reversed upon treatment with the inhibitor (Figure 5D). Quantitative analysis of immunofluorescence-positive areas drew consistent conclusions (Figure 5E,F). Moreover, identical results were obtained in primary alveolar macrophages; AMPK activation and autophagic flux were both suppressed in the presence of the inhibitor (Figure S4A,B). Immunohistochemical analysis corroborated these findings, showing that fibrotic areas in the β-glucan + BLM + Compound **C** group not only displayed characteristic collagen deposition and structural disruption but also a pronounced reduction in regional p-ULK1-positive cell clustering (Figure 5G,H). Both immunofluorescence and immunohistochemistry assays pointed toward that the activation of this signaling pathway was inhibited in the presence of the inhibitor. We then proceeded to investigate its effect on macrophage apoptosis. Flow cytometry with double staining indicated that the proportion of apoptotic cells in fibrotic lesions was elevated in the TGF-β + β-glucan + Compound **C** group compared with the untreated group (Figure 5I,J). Together, these results clearly demonstrate that the AMPK-mTOR-ULK1 axis constitutes the structural basis changing through which TI coordinates the autophagy–apoptosis balance.

### 3.6. Autophagy Inhibition Reverses the Protective Effects of TI In Vivo

**Figure 6:**
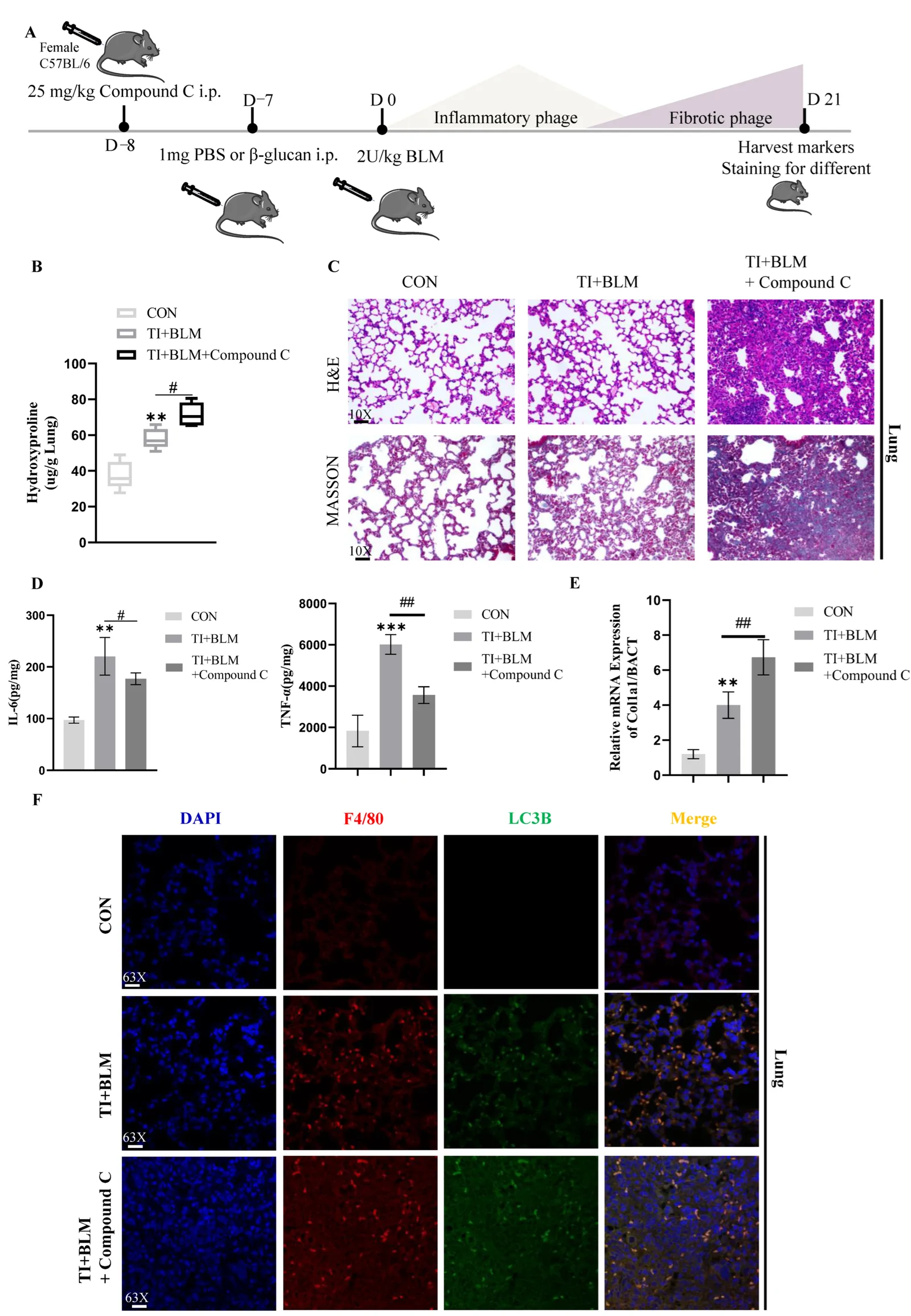
Autophagy inhibition reversed the protective effect of TI in the body. (**A**) In vivo model of pulmonary fibrosis treated with BLM + TI and AMPK inhibitor Compound **C**. (**B**) Hydroxyproline content in the lungs of mice in the control group, TI + BLM group, and TI + BLM + Compound **C** group. (**C**) Masson trichromatic staining and H&E staining of lung tissues in each group. (**D**) The content of TNF-α and IL-6 in bronchoalveolar lavage fluid of mice determined by ELISA. (**E**) Col1a1 mRNA expression in lung tissue quantified by qPCR. (**F**) Compared with the TI + BLM group, TI + BLM + Compound would reduce the co-localization of alveolar macrophages F4/80 and LC3B. Cell nucleus (blue), F4/80 (red), LC3B (green). Single dots correspond to individual mice. ** *p* < 0.01 and *** *p* < 0.001; paired Student’s *t*-test comparing BLM group and control group. ^#^ *p* < 0.05 and ^##^ *p* < 0.01; paired Student’s *t*-test comparing between TI + BLM group and TI + BLM + Compound **C** group.

To validate the specific role of autophagy in the mouse model of fibrosis, we conducted a series of in vivo experiments (Figure 6A). Following treatment with Compound **C** (an AMPK inhibitor) in combination with BLM and β-glucan, hydroxyproline assays revealed a significant increase in collagen content in lung tissues compared to mice treated with BLM and β-glucan alone (Figure 6B). H&E staining showed severe destruction of alveolar architecture and extensive inflammatory cell infiltration in the BLM group, while MASSON staining confirmed substantial blue collagen fiber deposition (Figure 6C). ELISA further indicated a reduction in the concentration of the key pro-inflammatory cytokine TNF-α and IL-6 in lung tissues from the BLM model (Figure 6D). We further employed qPCR to determine the transcriptional levels in mouse lung tissue, and the results showed that the inhibitor also affected collagen expression in mice (Figure 6E). Additionally, multicolor immunofluorescence revealed diminished spatial proximity between F4/80^+^ macrophages and the autophagy-related protein LC3B in fibrotic regions (Figure 6F). Together, these findings establish autophagy as an indispensable component through which TI exerts its protective effects against pulmonary fibrosis.

## 4. Discussion

IPF is an intractable pulmonary disease predominantly characterized by excessive extracellular matrix (ECM) accumulation, which triggers lung scarring, architectural destruction, dyspnea and ultimately death [23]. The progression of pulmonary fibrosis can be divided into two key phases: the early inflammatory phase and the late fibrotic phase. The early alveolar inflammatory phase is featured by prominent alveolar inflammation, wherein macrophages secrete inflammatory cytokines to eliminate pathogens and initiate inflammatory responses [24]. The subsequent fibrotic phase is dominated by pro-fibrotic M2 macrophages. As pro-fibrotic macrophage subsets, M2 macrophages secrete pro-fibrotic mediators including TGF-β to drive the proliferation and differentiation of fibroblasts, ultimately leading to excessive deposition of collagen and other extracellular matrix components and exacerbating pulmonary fibrosis [25]. We recognize the vital regulatory role of macrophages correlating without the fibrotic progression, yet in-depth investigations focusing on macrophage-driven fibrotic pathogenesis remain scarce. In addition, TI, a newly identified form of innate immune memory, represents a unique adaptive memory phenotype exclusive to innate immune cells such as macrophages, which elicits amplified immune responses against secondary infectious stimuli upon re-challenge [26]. Given that numerous patients with pulmonary fibrosis succumb to unregulated ECM deposition, exploring how TI-primed macrophages modulate pathological progression during the entire fibrotic cascade carries substantial scientific significance. Meanwhile, we also observed a certain discrepancy between the elevated levels of inflammatory cytokines induced by TI and the alleviative phenotype of pulmonary fibrosis detected in this model.

AMPK serves as a cellular energy sensor that initiates autophagy by directly phosphorylating and activating ULK1 or indirectly via mTOR inhibition [27]. In IPF, downregulated ubiquitin-specific peptidase 10 (USP10) has been shown to interact with Sirt6, promoting AMPK-associated with autophagy and attenuating fibrosis. USP10 suppresses the AKT/mTOR signaling axis correlating with Sirt6, thereby relieving mTOR associated with inhibition of autophagy [28]. AMPK activity influences macrophage functional states, and AMPK-regulated autophagy may help suppress polarization toward a pro-fibrotic M2 phenotype [29]. IPF lungs exhibit metabolic alterations such as enhanced glycolysis, and AMPK represents a key node for correcting such aberrant metabolism. By clearing dysfunctional mitochondria, AMPK activation helps maintain cellular homeostasis [23]. Although AMPK has been identified as a critical target for suppressing bleomycin-induced pulmonary fibrosis [30], the precise molecular mechanisms regulating AMPK in this context remain unclear. As an AMP-dependent protein kinase, AMPK is a central regulator of bioenergetic metabolism. It inhibits mTOR and enhances glycolysis [31], while modulating levels of key metabolites such as acetyl-CoA, thereby supplying substrates for histone modifications [32]. These pleiotropic functions underscore the importance of identifying upstream signals that directly regulate AMPK. Insights from oncology and related fields suggest that TI promotes AMPK phosphorylation, leading to enhanced mitophagy and reduced apoptosis. Both TI and AMPK signaling represent promising therapeutic targets for future interventions in pulmonary fibrosis.

Mitochondria are vital intracellular organelles that serve as the powerhouse of the cells responsible for energy production. During normal cellular metabolism, certain mitochondria become damaged or senescent, exhibiting impaired energy production and even releasing harmful substances such as reactive oxygen species (ROS). Under such circumstances, cells automatically recognize defective mitochondria and trigger mitophagy as an intrinsic self-protective mechanism [33]. Accumulation of damaged mitochondria induces massive secretion of inflammatory cytokines and ROS, which act as core signaling molecules driving fibroblast activation. Activated fibroblasts secrete abundant collagen, resulting in lung tissue stiffening and scar formation, thereby initiating and exacerbating pulmonary fibrosis [34]. Excessive clearance of mitochondria leads to insufficient mitochondrial pools and delayed energy supply, and cells eventually undergo death due to energy exhaustion, which likewise causes tissue injury and accelerates disease progression [35]. The lung is an essential organ for sustaining basic physiological activities; timely elimination of damaged mitochondria is required to preserve intact pulmonary function and maintain homeostatic balance. In addition, the establishment of TI relies on metabolic reprogramming [36]. In the microenvironment of IPF, a characteristic metabolic shift toward enhanced glycolysis not only meets cellular bioenergetic demands but also serves as the foundation for sustaining epigenetic memory associated with TI [23]. Nevertheless, further investigations are still required to clarify how mitophagy is balanced during the progression of pulmonary fibrosis. Furthermore, the precise role of mitophagy activation in the alleviation of pulmonary fibrosis remains the primary focus of follow-up investigations derived from this study.

Our above experiments revealed that mitophagy emerges along with the onset of cellular apoptosis, whereas TI alleviates macrophage apoptosis and maintains normal cellular homeostasis. Apoptosis refers to a genetically tightly regulated programmed cell death pathway [37]. The complicated regulatory network linking mitophagy and apoptosis bears profound research value in the field of pulmonary fibrosis. This study confirmed that TI inhibits apoptosis while facilitating mitophagy, yet the quantitative balance between mitophagy and apoptosis still merits further in-depth exploration. Additionally, one limitation of this study is the limited sample size. Multi-center research designs with larger sample cohorts can be adopted in future investigations for further verification of our conclusions.

AMPK and ULK1 are evolutionarily highly conserved serine/threonine protein kinases. The core function of AMPK is to sense intracellular energy status and sustain energy homeostasis, and it plays a pivotal role in maintaining mitochondrial balance. ULK1 is primarily responsible for initiating autophagy. Notably, AMPK acts as a critical upstream regulator of ULK1 [38,39]. In IPF, downregulated ubiquitin-specific peptidase 10 (USP10) has been shown to interact with Sirt6, promoting AMPK associated with autophagy and attenuating fibrosis. USP10 suppresses the AKT/mTOR signaling axis correlating with Sirt6, thereby relieving mTOR associated with inhibition of autophagy [28]. AMPK activity influences macrophage functional states, and AMPK-regulated autophagy may help suppress polarization toward a pro-fibrotic M2 phenotype [29]. Based on the above findings and diverse experimental approaches, we indicated that TI macrophages modulate mitophagy via the AMPK-ULK signaling cascade, thereby alleviating pulmonary fibrosis. Although β-glucan was applied to induce TI in alveolar macrophages in the current study [40], it remains an unresolved question whether distinct TI triggers exert such protective effects correlating with identical or divergent signaling pathways. In addition, Compound **C**, the inhibitor adopted in this experiment, may exert off-target effects, which renders the exact function of this signaling pathway in the present study unconfirmed. Moreover, this signaling pathway was identified based on bioinformatic analysis and the published literature. Knockout assays are still required to validate the stringent regulatory interactions between the signaling pathways, which constitutes a major limitation of this experiment and necessitates further experimental verification.

Following stimulation with TI inducers such as β-glucan, alveolar macrophages undergo substantial metabolic reprogramming. While our focus on alveolar macrophages is mechanistically justified, it may overlook potential contributions from other immune populations. The influence of TI on neutrophil extracellular trap formation, antigen presentation by dendritic cells, and lymphocyte polarization in fibrosis warrants dedicated investigation.

The purpose of the study is to elucidate the pivotal mechanism by which TI alleviates pulmonary fibrosis. Investigating the effects of TI on other subtypes of fibrosis or inflammatory diseases also bears great scientific significance. Intervening mitophagy and its downstream consequences including energy depletion and pro-inflammatory cytokine secretion may serve as an effective therapeutic strategy for ameliorating pulmonary fibrosis. Furthermore, autophagy targeting distinct organelles constitutes a sophisticated and profound scientific topic. Research in this field holds potential value for advancing precision medicine and developing therapeutic regimens against fibrotic disorders.

## 5. Conclusions

Autophagy is an important self-protection mechanism that is indispensable in patients with pulmonary fibrosis and is also crucial in TI. We assume that TI would enhance the protective effect against pulmonary fibrosis correlating with this mechanism. In this study, we establish that TI attenuates pulmonary fibrosis correlating with the AMPK signaling axis. Genetic or pharmacological inhibition of AMPK with Compound **C** was found to exacerbate fibrotic pathology. Furthermore, we indicate that TI reduces mitochondrial DNA release and promotes the co-localization of mitochondria with LC3B, indicative of enhanced mitophagy. Conversely, AMPK inhibition not only diminished the therapeutic benefits of TI but also increased cellular apoptosis. Collectively, our findings elucidate a previously unrecognized pathway correlating with which TI counteracts pulmonary fibrosis by improving mitochondrial quality control and suppressing apoptosis in alveolar macrophages. This work expands the functional scope of TI beyond infectious contexts and identifies metabolic regulation as a promising frontier for anti-fibrotic therapy. The mechanistic insights and therapeutic strategies emerging from this study warrant further investigation in both preclinical and clinical settings.

## Figures and Tables

**Table 1:** Primer sequences.

Gene	Sense (5′-3′)	Anti-Sense (5′-3′)
*COL1A1*	GCTCCTCTTAGGGGCCACT	CCACGTCTCACCATTGGGG
*ACTA2*	TGCTGACAGAGGCACCACTGAA	CAGTTGTACGTCCAGAGGCATAG
*ATG5*	CTTGCATCAAGTTCAGCTCTTCC	AAGTGAGCCTCAACCGCATCCT
*IL-6*	TAGTCCTTCCTACCCCAATTTCC	TTGGTCCTTAGCCACTCCTTC
*TNF-α*	CAGCAAGGGACAGCAGAGG	AGTATGTGAGAGGAAGAGAACC
*BACT*	GGCTGTATTCCCCTCCATCG	CCAGTTGGTAACAATGCCATGT

## Data Availability

The datasets generated for this study are available on request to the corresponding author. This study did not generate any additional resources.

## Supplementary Materials

The following supporting information can be downloaded at https://www.mdpi.com/article/10.3390/biology15161366/s1.

## References

[1] Ochando J., Mulder W.J.M., Madsen J.C., Netea M.G., Duivenvoorden R. (2023). Trained immunity—Basic concepts and contributions to immunopathology. Nat. Rev. Nephrol..

[2] Vuscan P., Kischkel B., Joosten L.A.B., Netea M.G. (2024). Trained immunity: General and emerging concepts. Immunol. Rev..

[3] Ziogas A., Bruno M., van der Meel R., Mulder W.J.M., Netea M.G. (2023). Trained immunity: Target for prophylaxis and therapy. Cell Host Microbe.

[4] Gao F., Pan L., Liu W., Chen J., Wang Y., Li Y., Liu Y., Hua Y., Li R., Zhang T. (2025). Idiopathic pulmonary fibrosis microenvironment: Novel mechanisms and research directions. Int. Immunopharmacol..

[5] Zhang M., Zhang J., Hu H., Zhou Y., Lin Z., Jing H., Sun B. (2024). Multiomic analysis of monocyte-derived alveolar macrophages in idiopathic pulmonary fibrosis. J. Transl. Med..

[6] Li S., Liu G., Gu M., Li Y., Li Y., Ji Z., Li K., Wang Y., Zhai H., Wang Y. (2022). A novel therapeutic approach for IPF: Based on the “Autophagy—Apoptosis” balance regulation of Zukamu Granules in alveolar macrophages. J. Ethnopharmacol..

[7] Aegerter H., Lambrecht B.N., Jakubzick C.V. (2022). Biology of lung macrophages in health and disease. Immunity.

[8] Vargas J.N.S., Hamasaki M., Kawabata T., Youle R.J., Yoshimori T. (2023). The mechanisms and roles of selective autophagy in mammals. Nat. Rev. Mol. Cell Biol..

[9] Lizama B.N., Chu C.T. (2021). Neuronal autophagy and mitophagy in Parkinson’s disease. Mol. Asp. Med..

[10] Lu Y., Li Z., Zhang S., Zhang T., Liu Y., Zhang L. (2023). Cellular mitophagy: Mechanism, roles in diseases and small molecule pharmacological regulation. Theranostics.

[11] Jeong S., Yang K., Lee Y.J., Park J.W., Park E.M., Kang J.L. (2025). Gas6 induces AIM to suppress acute lung injury in mice by inhibiting NLRP3 inflammasome activation and inducing autophagy. Front. Immunol..

[12] Wang J., Yang K., Liu C., Wang F., Liang X., Wu J., Hong S., Yin X., Zhang X. (2025). H3 relaxin ameliorates mitochondrial quality control and apoptosis in cardiomyocytes of type 2 diabetic rats via activation of the AMPK pathway. Int. Immunopharmacol..

[13] Yang C., Rubin L., Yu X., Lazarovici P., Zheng W. (2024). Preclinical evidence using synthetic compounds and natural products indicates that AMPK represents a potential pharmacological target for the therapy of pulmonary diseases. Med. Res. Rev..

[14] Nakazawa S., Hou J., Kato M., Togo S., Arai Y., Motomura H., Kurata K., Sueyasu T., Hirakawa H., Ochi Y. (2025). Allicin induced AMPK signaling attenuated Smad3 pathway mediated lung fibrosis. Sci. Rep..

[15] Zhou Y., Wang Y., Wang M., Li B., Xu K., Pan X., Li Z., Wang Q., He W., Pang J. (2025). CAMKK2 restored mitochondrial dynamics homeostasis to alleviate pulmonary fibrosis via AMPK/PGC-1α signaling pathway in lung fibroblasts. Mol. Med..

[16] Sun Z., Qu J., Xia X., Pan Y., Liu X., Liang H., Dou H., Hou Y. (2021). 17β-Estradiol promotes LC3B-associated phagocytosis in trained immunity of female mice against sepsis. Int. J. Biol. Sci..

[17] Sun Z., Ji Z., He W., Duan R., Qu J., Yu G. (2024). Lactate accumulation induced by Akt2-PDK1 signaling promotes pulmonary fibrosis. FASEB J..

[18] Liu X., Chhipa R.R., Nakano I., Dasgupta B. (2014). The AMPK inhibitor compound C is a potent AMPK-independent antiglioma agent. Mol. Cancer Ther..

[19] Sun Z., He W., Meng H., Ji Z., Qu J., Yu G. (2024). Lactate activates ER stress to promote alveolar epithelial cells apoptosis in pulmonary fibrosis. Respir. Res..

[20] Arts R.J.W., Carvalho A., La Rocca C., Palma C., Rodrigues F., Silvestre R., Kleinnijenhuis J., Lachmandas E., Gonçalves L.G., Belinha A. (2016). Immunometabolic Pathways in BCG-Induced Trained Immunity. Cell Rep..

[21] Yao M., Zhou J., Mei J., Gao C., Ding P., Li G., Zhang C., Li Z., Gao J. (2025). Trained Immunity in Health and Disease. MedComm.

[22] Wei Y., Guo H., Yang X., Tang X.X. (2026). Macrophage-to-Myofibroblast Transdifferentiation Contributes to Pulmonary Fibrosis via the MERTK-SPP1-SRC-TKS5 Signaling Axis. Adv. Sci..

[23] Li G., Huo Y., Pan X., Jia N., Wu X., Wu X., Wang F., Du Q. (2025). Targeting the immunometabolism interface: A novel strategy for IPF therapy. Pulm. Pharmacol. Ther..

[24] Bigot J., Legendre R., Hamroune J., Jacques S., Le Gars M., Millet N., Guillot L., Corvol H., Hennequin C., Guitard J. (2025). The epigenomic landscape of bronchial epithelial cells reveals the establishment of trained immunity. Genes Immun..

[25] Zuo L., Liu Q., Ye D., Fan J., Wu L. (2025). Idiopathic Pulmonary Fibrosis: Cellular Heterogeneity, Mechanisms, and Therapeutic Implications. MedComm.

[26] Schlüter T., van Elsas Y., Priem B., Ziogas A., Netea M.G. (2025). Trained immunity: Induction of an inflammatory memory in disease. Cell Res..

[27] Trefts E., Shaw R.J. (2021). AMPK: Restoring metabolic homeostasis over space and time. Mol. Cell.

[28] Mao S., Yu N., Wang W., Mao Y., Du Y., Zhao Q., Gu X., Kang J. (2025). Ubiquitin-specific peptidase 10 attenuates bleomycin-induced pulmonary fibrosis via modulating autophagy depending on sirtuin 6-mediated AKT/mTOR. Cell Biol. Toxicol..

[29] Cui Y., Chen J., Zhang Z., Shi H., Sun W., Yi Q. (2023). The role of AMPK in macrophage metabolism, function and polarisation. J. Transl. Med..

[30] Wang Y., Wang X., Du C., Wang Z., Wang J., Zhou N., Wang B., Tan K., Fan Y., Cao P. (2024). Glycolysis and beyond in glucose metabolism: Exploring pulmonary fibrosis at the metabolic crossroads. Front. Endocrinol..

[31] Hsu C.C., Peng D., Cai Z., Lin H.K. (2022). AMPK signaling and its targeting in cancer progression and treatment. Semin. Cancer Biol..

[32] Steinberg G.R., Hardie D.G. (2023). New insights into activation and function of the AMPK. Nat. Rev. Mol. Cell Biol..

[33] Wang Q., Sun Y., Li T.Y., Auwerx J. (2026). Mitophagy in the pathogenesis and management of disease. Cell Res..

[34] Sun Z., Ji Z., Meng H., He W., Li B., Pan X., Zhou Y., Yu G. (2024). Lactate facilitated mitochondrial fission-derived ROS to promote pulmonary fibrosis via ERK/DRP-1 signaling. J. Transl. Med..

[35] Pokharel M.D., Garcia-Flores A., Marciano D., Franco M.C., Fineman J.R., Aggarwal S., Wang T., Black S.M. (2024). Mitochondrial network dynamics in pulmonary disease: Bridging the gap between inflammation, oxidative stress, and bioenergetics. Redox Biol..

[36] Fang H., Xiong Y., Fu B., Wu H. (2025). Targeting alveolar macrophages in tuberculosis: Exploiting trained immunity for novel therapeutic approaches. Int. Immunopharmacol..

[37] Li Z., Li Y., Mao N., Gao X., Xu H., Cai W., Li T. (2026). Ceramide-Driven Mechanisms in Pulmonary Fibrosis. Metabolites.

[38] Iorio R., Celenza G., Petricca S. (2021). Mitophagy: Molecular Mechanisms, New Concepts on Parkin Activation and the Emerging Role of AMPK/ULK1 Axis. Cells.

[39] Chen X., Shi X., Yu Z., Ma X. (2023). High-intensity interval training in breast cancer patients: A systematic review and meta-analysis. Cancer Med..

[40] Li P., Liu M., Liu Y., Guo J., Jiang J., Wang G., Cao C., Wang X., Qu J., Sun Z. (2025). Recent Advances of Trained immunity in Macrophages. Int. J. Biol. Sci..

