# Supplementary material for: Trained Immunity Attenuates Bleomycin-Induced Pulmonary Fibrosis by Promoting AMPK-Mediated Autophagy in Alveolar Macrophages
Source: Biology (Basel). 2026 Aug 11;15(16):1366. doi: 10.3390/biology15161366 (PMC13509561; doi:10.3390/biology15161366)
Supplement: Supplementary file 1 [file biology-15-01366-s001.zip › biology-4435939-Supplementary figures.pdf]

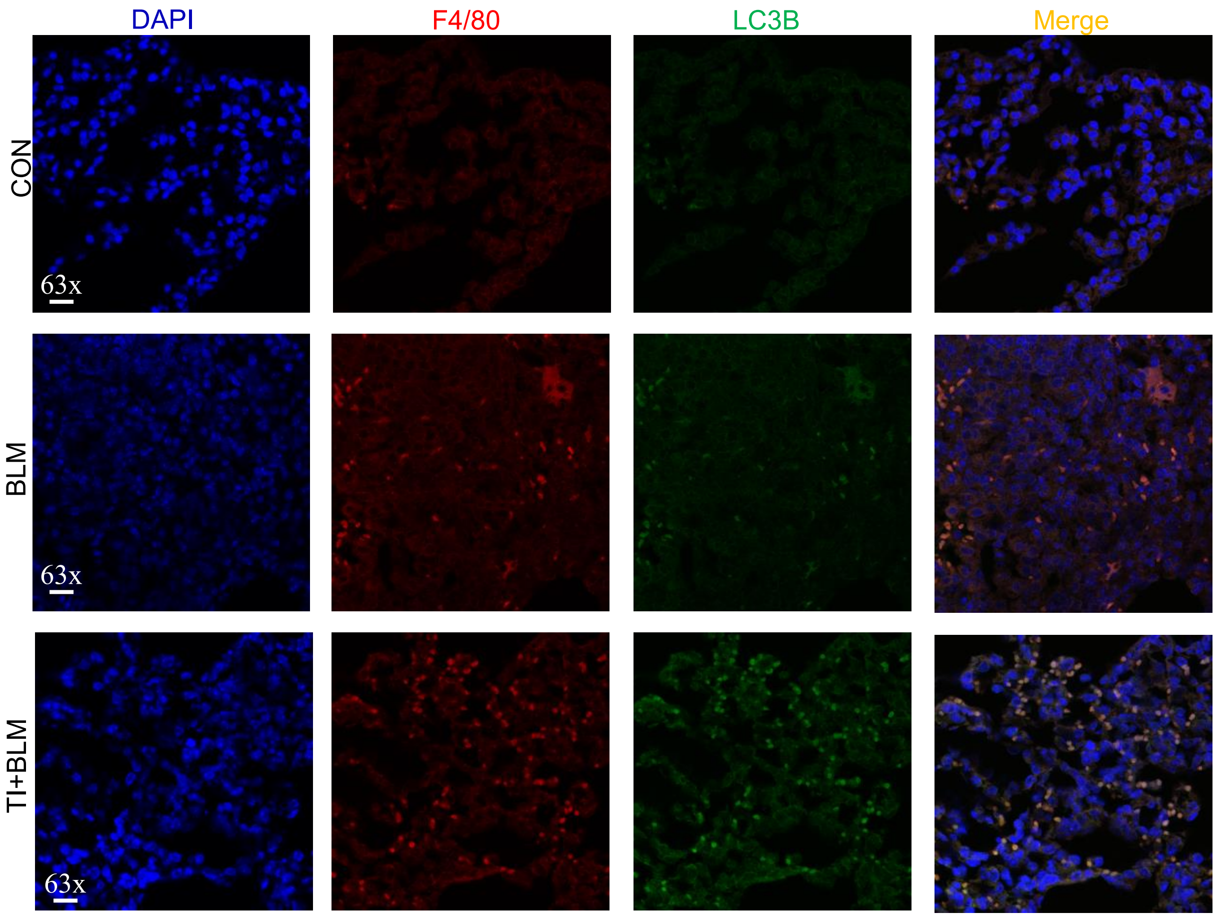

Figure S1. TI can alleviate pulmonary fibrosis induced by BLM. The co-localization of alveolar macrophages F4/80 and autophagy LC3B in mouse lung tissue was shown by immunofluorescence (n = 3).

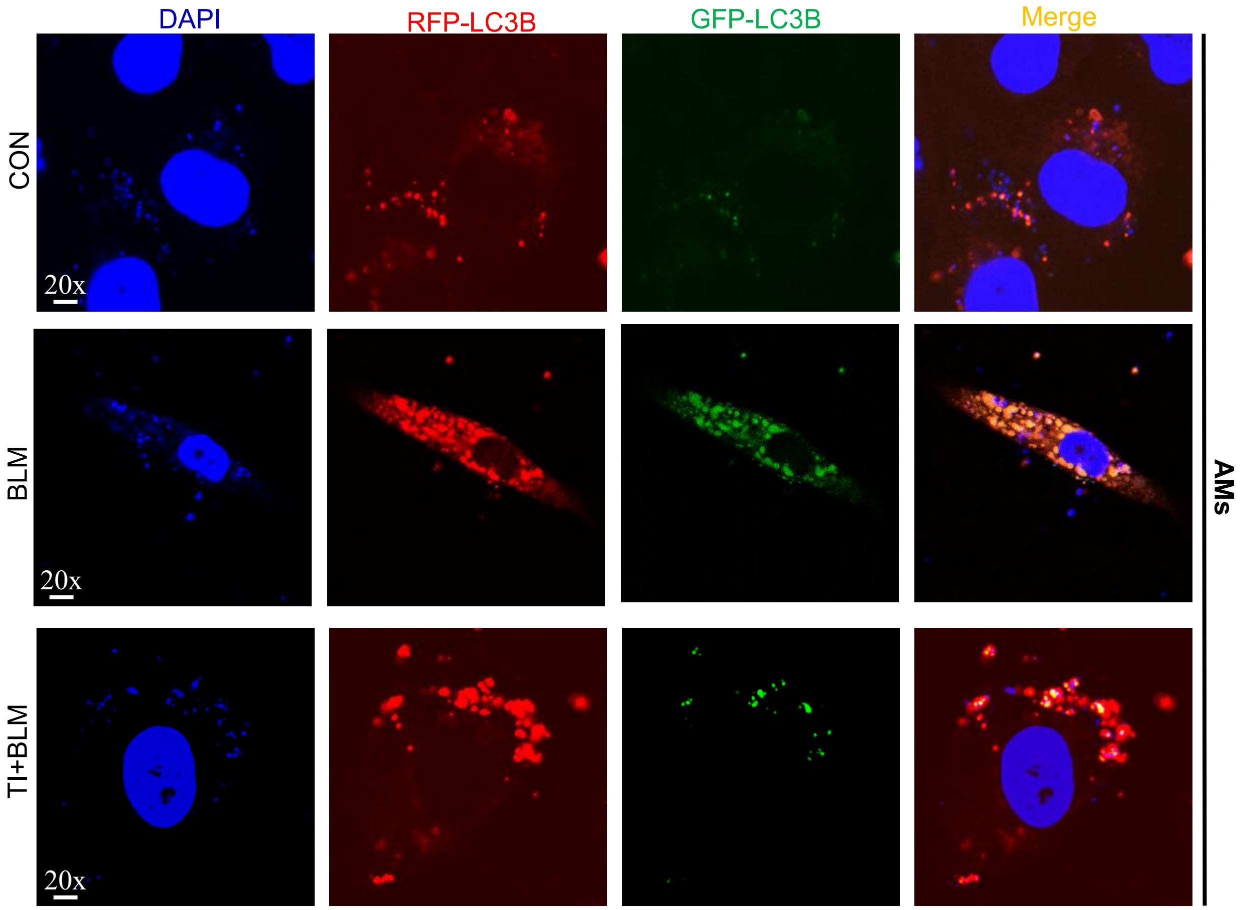

Figure S2. TI promotes mitochondrial autophagy in both vivo and vitro and alleviates apoptosis of alveolar macrophages. Using the dual-fluorescence GFP-LC3B (green) and RFP-LC3B (red) labeling system, GFP-LC3B puncta (green) represent autophagosomes, while RFP-LC3B (red) retains its fluorescence in acidic environments (autolysosomes). The ratio of GFP to RFP signals reflects the efficiency of autophagic flux; merged images (Merge) demonstrate the co-localization of the two fluorescent signals. Nuclei were counterstained with DAPI (blue).

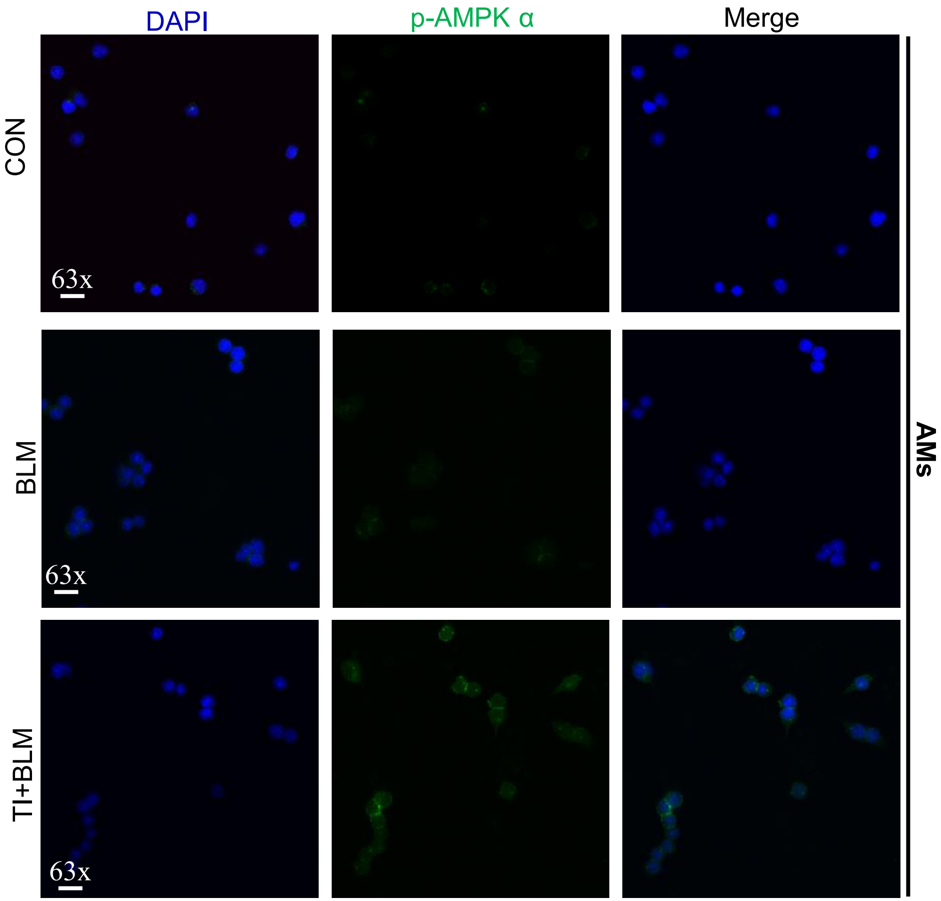

Figure S3. TI mainly activates autophagy through the AMPK-mTOR-ULK signaling pathway to in-hibit pulmonary fibrosis. Immunofluorescence showed that the expression of AMPK was significant after TI+TGF-β treatment, Nucleus (blue), p-AMPK(green).

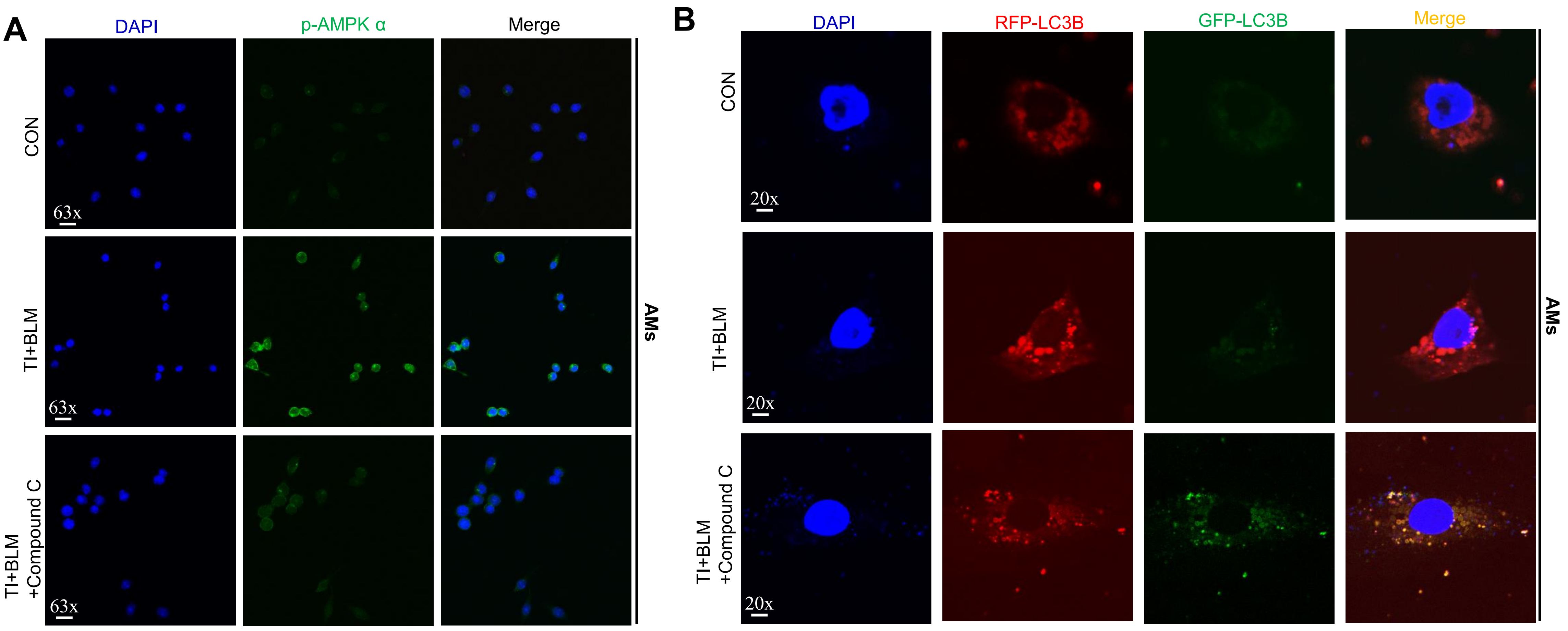

Figure S4. Phosphorylation of AMPK-mTOR-ULK1 pathway proteins affects macrophage apoptosis caused by TI. (A) Immunofluorescence showed that the expression of AMPK was significant after TI+TGF-β treatment, Nucleus (blue), p-AMPK(green). (B) Using the dual-fluorescence GFP-LC3B (green) and RFP-LC3B (red) labeling system, GFP-LC3B puncta (green) represent autophagosomes, while RFP-LC3B (red) retains its fluorescence in acidic environments (autolysosomes). The ratio of GFP to RFP signals reflects the efficiency of autophagic flux; merged images (Merge) demonstrate the co-localization of the two fluorescent signals. Nuclei were counterstained with DAPI (blue).

A

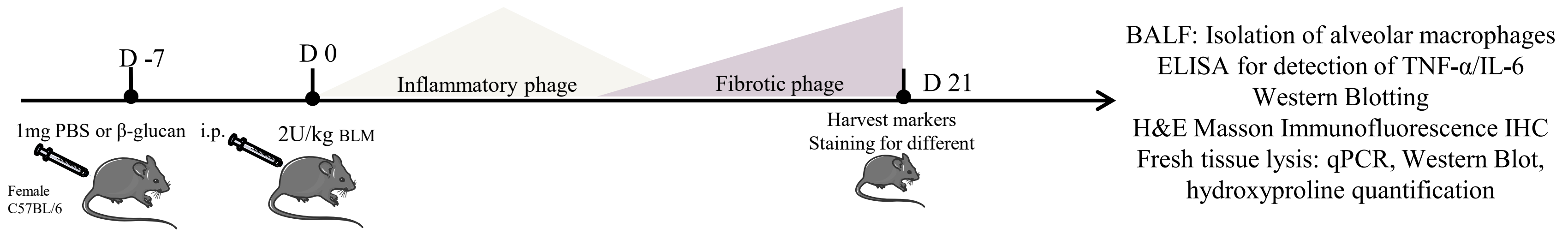

B

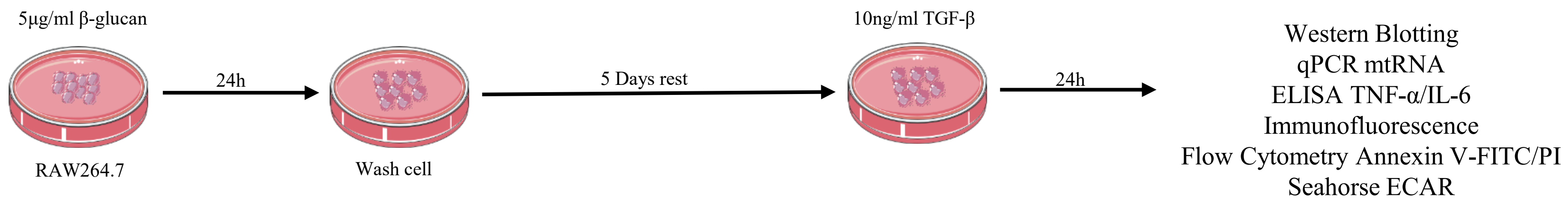

Figure S5. A: The in vivo experiments of this study utilized C57BL/6 mice to establish a pulmonary fibrosis model induced by bleomycin (BLM). The experiments were divided into three groups: control group (CON), BLM group, and TI + BLM group. The mouse TI model was established by intraperitoneal injection of β-glucan, and Compound C was used for pathway inhibition. After modeling, the mice were continuously observed for 21 days and their body weight was recorded. At the end of the experiment, bronchoalveolar lavage fluid (BALF) and lung tissues were collected, and the degree of pulmonary fibrosis, autophagy level, and inflammatory response in the mouse lung tissues were comprehensively evaluated through pathological staining, molecular biology detection, and other methods. B: RAW264.7 mouse macrophages were used as the model for in vitro experiments. Firstly, the training immunity (TI) model was established by using β-glucan induction. Then, the fibrotic injury model was constructed by stimulating with TGF-β, and the AMPK inhibitor Compound C intervention group was set up for mechanism verification. The experiment was divided into four groups: control group (CON), TGF-β group, and TI + TGF-β group. Finally, cell samples were collected and various techniques such as Western blotting (WB), immunofluorescence, cell metabolism analysis, flow cytometry, qPCR and ELISA were used to detect cell fibrosis, autophagy, metabolism and inflammation-related indicators.
